# Predictive Impact of The Inflammation-based Indices in Uveal Melanoma Liver Metastases Treated with Transarterial Hepatic Chemoperfusion

**DOI:** 10.2478/raon-2021-0027

**Published:** 2021-05-31

**Authors:** Johannes M. Ludwig, Johannes Haubold, Sebastian Bauer, Heike Richly, Jens T. Siveke, Julia Wimmer, Lale Umutlu, Benedikt M. Schaarschmidt, Jens M. Theysohn

**Affiliations:** 1 Department of Diagnostic and Interventional Radiology and Neuroradiology, University Hospital Essen, University of Duisburg-Essen, Essen, Germany; 2 Department of Medical Oncology, Sarcoma Center, West German Cancer Center, University of Duisburg-Essen, Essen, Germany; 3 Institute for Developmental Cancer Therapeutics, West German Cancer Center, University Hospital Essen, Essen, Germany; 4 Division of Solid Tumor Translational Oncology, German Cancer Consortium (DKTK, partner site Essen) and German Cancer Research Center, DKFZ, Heidelberg, Germany

**Keywords:** uveal melanoma, liver metastases, transarterial hepatic chemoperfusion, melphalan, inflammatory markers

## Abstract

**Background:**

The aim of the study was to evaluate pretreatment inflammatory markers as prognostic factors in patients with unresectable uveal melanoma liver metastases treated with transarterial hepatic chemoperfusion.

**Patients and methods:**

54 patients (44% male, median age: 61 years) were retrospectively assessed. A median of 3 (range: 1–11) treatment sessions were performed with melphalan (92%) or fotemustin (8%). Inflammatory indices were calculated as follows: neutrophils/nl to lymphocytes/nl ratio (NLR), systemic immune-inflammation index ([platelets/nl × neutrophils/nl]/[lymphocytes/nl]; SII), and platelets/nl to lymphocytes/nl ratio (PLR). The cut-off for dichotomization purposes was set at the median (inflammatory indices, hepatic tumor burden) or the upper level of normal. Kaplan Meier analysis was performed for median overall survival (OS) in months, and Cox proportional hazard model for uni(UVA) and multivariate (MVA) hazard ratio (HR, 95%CI) analyses were performed.

**Results:**

Median OS of the study cohort was 7.7 (6.3–10.9) months. In UVA OS was prolonged for low C reactive protein (CRP) (13.5 *vs*. 5.2; p = 0.0005), low SII (10.8 *vs*. 5.6; p = 0.0005), low NLR (11.1 *vs*. 6.3; p = 0.0045), low aspartate aminotransferase (AST) (11.5 *vs*. 5.6; p = 0.015), alanine aminotransferases (ALT) (11.5 *vs*. 5.6; p = 0.01), and tumor burden ≦ 50% (8.2 *vs*. 4.8; p = 0.007). MVA confirmed low CRP (HR: 0.29, 0.11–0.7; p = 0.005), low SII (HR: 0.19, 0.11–0.7; p = 0.008), and low ALT (HR: 0.13, 0.02–0.63; p = 0.011) as independent predictors for prolonged OS. Patients with ≦ 1, 2, 3 elevated significant MVA-factors survived a median of 14.9, 7.7, and 3.9 months, respectively (p = 0.0001).

**Conclusions:**

Pretreatment inflammatory markers (CRP, SII) and AST were independent prognostic survival markers in patients with uveal melanoma liver metastases treated with transarterial hepatic chemoperfusion. A combination of factors may help to identify patients potentially benefitting from treatment.

## Introduction

Uveal melanoma is the most common primary ocular malignancy in adults accounting for around 5% of all melanomas.^1^, ^2^ Although local tumors are usually treated aggressively, about 50% of all patients will eventually develop metastases, with in 60.5% of cases involvement of the liver at the time of diagnosis of metastatic disease.^3^ In patients with predominant and unresectable hepatic disease, we routinely perform transarterial hepatic chemoperfusion (THC) as a palliative treatment option demonstrating prolonged progression-free survival and fewer hematological severe adverse events compared to intravenous chemotherapy.^4^

Setting expectations for treatment benefit and life expectancy is crucial for informed clinical decision-making and may guide patients and their families to set expectations. To date, few pretreatment prognostic factors on treatment outcomes, including but not limited to tumor burden, lactate dehydrogenase (LDH), and gamma-glutamyl transferase (GGT) serum values have been reported.^5^, ^6^

The role of inflammation has long been acknowledged as a hallmark during cancerogenesis and tumor progression in malignant disease.^7^, ^8^ In the context of tumor-associated inflammation, the systemic inflammatory response is linked with poorer outcomes and as of significant prognostic relevance in various cancer types.^9^, ^10^ This systemic inflammatory response is usually measured in the peripheral blood with numbers of differential blood cell counts (lymphocytes, neutrophils, platelets) as well as serum proteins (C-reactive protein, albumin). Here, the inflammation-based indices of differential cell counts, the neutrophil to lymphocyte ratio (NLR), the platelet to lymphocyte ratio (PLR), and the systemic immune-inflammation index (SII) have been proven as significant prognostic factors in several cancer types.^11^

The purpose of this study was to evaluate inflammatory markers routinely assessed before THC as pretreatment prognostic factors in patients with unresectable uveal melanoma liver metastases.

### Patients and methods

#### Study population

This study is a retrospective single-center database analysis that has been approved by the local institutional review board with waived informed consent (IRB#: 20-9799-BO). All procedures performed in studies involving human participants were in accordance with the ethical standards of the institutional and/or national research committee and with the 1964 Helsinki declaration and its later amendments or comparable ethical standards. The decision to perform transarterial chemoperfusion was based on multidisciplinary tumor board meetings.

Fifty-four consecutive patients first treated in the years 2014 and 2015 were included in this study. Inclusion criteria were as follows: I) At least 18-years of age, II) imaging or biopsy-proven uveal melanoma liver metastases, and III) treatment of liver metastases with THC. Patient data were obtained from the medical record system, including disease history and laboratory testing results before treatment.

#### Treatment

Transarterial hepatic chemoperfusion was performed by gaining access via the femoral artery by inserting a 5 Fr catheter sheath and placing a micro-catheter into the hepatic arteries, either in the proper hepatic artery or consecutively in the left, right, and/or accessory hepatic arteries. Chemoperfusion of the liver was performed for 45–60 minutes. If the dose was infused into two lobes, a median of 30% for the left and 70% for the right lobe of the total dose were administered. All patients started with 40 mg of melphalan. In case of progression, either the melphalan dose was escalated (45 mg, 50 mg), or the chemotherapeutic agent was switched to fotemustin.

#### Data collection

Laboratory blood test values were measured within thirty days before the first THC: Alanine aminotransferases (ALT; normal: < 35 U/L), aspartate aminotransferase (AST, normal: < 35 U/L), alkaline phosphatase (AP, normal: 20–100 U/L), gammaglutamyl transpeptidase (GGT, normal <35 U/L), lactate dehydrogenase (LDH, normal 120–247 U/L). Furthermore, absolute neutrophils (ANC, normal: 1.7–6.2/nl), lymphocytes (ALC, normal: 1.0–3.4/nl), and platelets (APC, normal: 180–380/nl) counts were obtained for calculating inflammatory indices. The neutrophil to lymphocyte ratio (NLR) was defined as the ratio of ANC/ALC, the platelet to lymphocyte ratio (PLR) as the ratio of APC/ALC, and the SII as the platelet count x ANC/ALC. For dichotomization purposes, the cut-off values were set at the upper level of normal (ULN) for laboratory values, at the median for inflammatory indices and tumor burden, or according to categorical status. Analysis of albumin and bilirubin have not been performed due to inconsistent and low numbers of reporting. The date of death was obtained regardless of etiology.

#### Statistics

The Kaplan-Meier method (log-rank test) was applied for estimating the overall survival (OS) with 95% confidence intervals (95%CI). Uni- (UVA) & multivariate (MVA) analyses for determining the hazard ratios (HR), including the 95% CI were calculated utilizing the Cox proportional hazards model. Factors statistically significant in UVA were included in MVA. For correlation analysis, the Spearman analysis was performed. Contingency testing was calculated using Pearson’s chi-squared test. Calculations were performed using JMP Pro 13.2.1 for Windows (SAS Institute Inc.). P-values < 0.05 were considered statistically significant.

## Results

### Patient baseline and treatment characteristics

The study consisted of fifty-four patients (44% male, 100% Caucasians, median age: 61 years; range: 26–81 years). Death was recorded in 42 patients, and 12 patients were lost to follow-up with a median follow-up of 15.8 months (95% CI: 2.3–24 months). The median time between primary diagnosis and occurrence of hepatic metastases was 24.4 months (range: 0–122 months). The first THC session was performed after a median of 4.6 months following diagnosis of liver metastases (range: 0.3– 38.7 months) with a median of 3 (range: 1–11) THC sessions/patient. The median time between treatments was 1.6 months (range 0.9 – 6.2). In total, 198 THC sessions were performed with melphalan in 186 (median: 40 mg, range: 40–50 mg) and fotemustin in 12 (median: 188 mg, range: 160–208 mg) cases. Eighteen patients (33%) received subsequent therapy after the last THC with systemic therapy in 13 Patients (8 patients received immune checkpoint inhibitors), radioembolization of liver metastases in 2 patients, and three patients received palliative external beam radiation of extrahepatic lesions for symptom control. Additional patient baseline characteristics are presented in Table 1.

**Table 1:** Overview of patient baseline characteristics

Characteristics	Number of patients (%) / median values
Total number of patients	54
Gender (male).	24 (44%)
Median age in years at 1^st^ THC (range)	61 (26–81)
**Prior systemic/liver-directed therapies**	
Prior systemic therapy	29 (53.7%)
Sorafenib	25 (46.3)
MEK and PKC inhibitors	3 (5.6%)
Ipilimumab	1 (1.9%)
Conventional chemotherapy	5 (9%)
Prior liver resection	4 (7.4%)
Prior ablation	1 (1.9%)
Further therapy after last transarterial chemoperfusion	18 (33.3%)
Limited extrahepatic metastases at the time of 1^st^ THC	20 (37%)
Median maximal tumor size in cm (range)	5.9 (1.3–19.8)
**Lobar tumor involvement**	
Bilobar	54 (96.4%)
Unilobar	2 (3.6%)
**Hepatic tumor burden**	
0–25%	23 (46%)
> 25–50%	12 (24%)
> 50–75%	9 (18%)
> 75%	6 (12%)
**ECOG**	
0	43 (80%)
1	6 (11%)
2	2 (4%)
Unknown	3 (6%)
**Karnofsky Index**	
100	4 (7%)
90	33(61%)
80	7 (13%)
< 80	3 (6%)
Unknown	7 (13%)

MEK = mitogen-activated protein kinase kinase enzymes MEK1 and/or MEK2; PKC = protein kinase C; THC = transarterial hepatic chemoperfusion

#### Survival analysis

**Figure 1:**
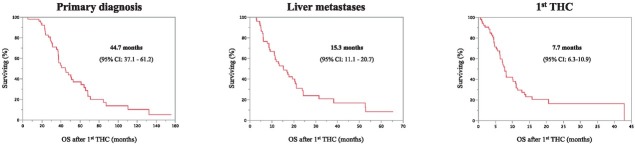
Overall survival of the entire study cohort. Estimated overall survival (OS) after diagnosis of primary, diagnosis of liver metastases, and after 1^st^ transarterial chemoperfusion (THC).

Following the diagnosis of primary tumor median survival of all patients was estimated to be 44.7 months (95% CI: 37.1–61.2). After the diagnosis of liver metastases, the median overall survival of 15.3 months (95% CI: 11.1–20.7) was observed. Median OS following first THC therapy was 7.7 months (95% CI: 6.3–10.9) (Figure 1). 6 months, 1- and 2 years survival rates were 67.4% (95% CI: 53.7–78.8%), 29.5% (95% CI: 18.7–43.8), and 16.5% (95%CI: 8.0–31.7) respectively.

#### Inflammatory prognostic factors

Median absolute cell counts were 5.13/nl for neutrophils (interquartile range [IQR]: 3.11–6.37), 1.36/ nl for lymphocytes (IQR: 1.02–1.71), and 267/nl for platelets (IQR: 209–346). When dichotomized at the median and upper level of normal, absolute cell counts were not significant regarding OS (p>0.05). Median values of inflammatory indices for the study cohort were 3.58 for NLR (IQR: 2.43–5.04), 1076 for SII (IQR: 539–1645), and 208.8 for PLR (IQR: 151.2–278). In Spearman’s analysis, the correlation between inflammatory markers ranged from very good (SII & NLR, Spearman’s ρ = 0.92) to good (SII & PLR, Spearman’s ρ = 0.72; PLR & NLR, Spearman’s ρ = 0.66). Decreased NLR and SII, as well as non-elevated C reactive protein (CRP), were both associated with more prolonged median OS, whereas PLR was not (Table 2).

**Table 2:** Uni- and multivariate analysis of overall survival (OS)

		Median (95OS % in CI) months	Univariate analysis		Multivariate analysis	
Subgroups			HR (95% CI)	p-value	HR (95% CI)	p-value
NLR	≦ median (3.58) > median (3.58)	11.1 (7.1–20.6) 6.3 (3.5–7.8)	0.39 (0.2–0.75) 1	**0.0045**	0.73 (0.25–2.2) 1	0.57
SII	≦ median (1076) > median (1076)	10.8 (7,2–20.6) 5.6 (3.4 – 7.7)	0.33 (0.17 – 0.65) 1	**0.0013**	0.19 (0.11–0.7) 1	**0.008**
PLR	≦ median (203.8) > median (203.8)	8.2 (5.6–15.8) 7.5 (4.7–11.1)	0.69 (0.37–1.27) 1	0.23	- -	**-**
CRP	normal > ULN	13.5 (7.2–20.6) 5.3 (3.9–7.8)	0.3 (0.15–0.6) 1	**0.0005**	0.29 (0.11–0.7) 1	**0.005**
Neutrophils	normal > ULN	8.2 (6.4–11.5) 6.3 (3.9–11.1)	0.6 (0.3–1.28) 1	0.18	- -	-
Thrombocytes	normal > ULN	8.2 (63–11.1) 7.5 (0.64–13.5)	0.8 (0.35–2.13) 1	0.62	- -	
LDH	≦ ULN >ULN	12.8 (7.2–20.6) 7 (4.8–8.2)	0.54 (0.23–1.11) 1	0.1	- -	-
AST	≦ ULN > ULN	11.5 (7.2–20.6) 5.6 (4.5–8.2)	0.45 (0.22–0.85) 1	**0.015**	0.34 (0.07–1.44) 1	0.15
ALT	≦ ULN > ULN	11.5 (7.5–15.8) 5.6 (4.2–7.8)	0.43 (0.2–0.8) 1	**0.01**	0.13 (0.02–0.63) 1	**0.011**
GGT	≦ ULN > ULN	10.9 (7.2–15.8) 7.13 (5.3–10.2)	0.94 (0.32–2.2) 1	0.9	- -	**-**
AP	≦ ULN > ULN	10.94 (4.8–20.6) 6.3 (3.4–10.1)	0.54 (0.22–1.26) 1	0.15	– -	**-**
Hepatic tumor burden	50% ≦ > 50%	8.2 (7.12–11.5) 4.8 (1.2–7.8)	0.36 (0.18–0.74) 1	**0.007**	0.5 (0.17–1.6) 1	0.24
Prior systemic treatment	Yes No	8.2 (7.5–11.5) 6.3 (4.5–11.1)	0.78 (0.42–1.45) 1	0.43	- -	-
Extrahepatic metastases	No Yes	7.12 (4.6–10.1) 10.2 (6.3–11.1)	1.1 (0.59–2.17) 1	0.75	- -	-

ALT = alanine aminotransferase; AP = alkaline phosphatase; AST = aspartate aminotransferase; CRP = C-reactive protein; GGT = gamma-glutamyl transpeptidase; HR = hazard ratio; LDH = lactate dehydrogenase; NLR = neutrophil to lymphocyte ratio; PLR = platelet to lymphocyte ratio; SII = systemic immune-inflammation index; ULN = upper level of normal

#### Non-inflammatory prognostic factors

Patients with serum values of the liver enzymes ALT and AST within the normal range had prolonged overall survival in contrast to AP and GGT. Aside from laboratory markers, the tumor burden was identified as a significant factor with patients with ≤ 50% hepatic tumor burden doing significantly better. In contrast, pretreatment status (yes *vs*. no; p = 0.18), presence of limited extrahepatic disease not considered life-limiting compared to liver metastases (yes *vs*. no; p = 0.3), ECOG Status (ECOG 0 *vs*. >= 1; p = 0.99), and Karnofsky Index (100–90% *vs*. < 90%; p = 0.44) were not significant (Table 2). Of note, patients who received treatment after last chemoperfusion showed a significant longer survival (13.9 *vs*. 7.2 months, p = 0.01; HR: 0.41; 95% CI: 0.21–0.83; p = 0.009). There was no significant difference regarding the SII between the patients with and without further treatment in contingency testing (p = 0.11).

#### Results from the multivariate analysis

In multivariate analysis, the overall strongest predictor with the lowest hazard ratio for values below the median was found for ALT (HR: 0.13; p = 0.011). SII proved as the strongest independent inflammatory predictor with a hazard ratio of 0.19 (p = 0.008) followed by CRP (HR: 0.29, p = 0.005). NLR and GOT were not identified as independent predictors for overall survival in this study population.

#### Scoring with significant factors from the multivariate analysis

Yet, as none of the identified independent predictors for overall survival was clearly stronger than the others, an additive scoring was performed to test for an additional predictive value of significant parameters (Figure 3). Patients with an elevation of none to one elevated parameter (SII, CRP, or ALT) survived significantly longer with a median overall survival of 14.9 months (95% CI: 10.1-0.0) compared to patients with two (6.7 months, 95% CI: 4.5–8.2) and all three parameters elevated (3.9 months, 95% CI: 1.15–6.3); p < 0.0001. Performed univariate analysis demonstrated a statistical difference between each group (Table 3).

**Figure 2:**
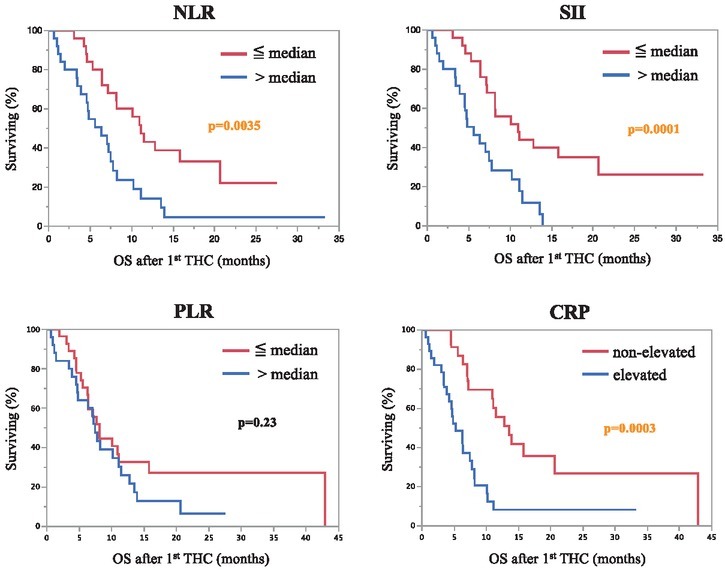
Pretreatment inflammation-based markers predict overall survival. Overall survival is stratified for low (≦ median) *vs*. high (> median) neutrophil to lymphocyte ratio (NLR), systemic immune-inflammation index (SII), platelet to lymphocyte ratio (PLR), and C-reactive protein (CRP).

**Figure 3:**
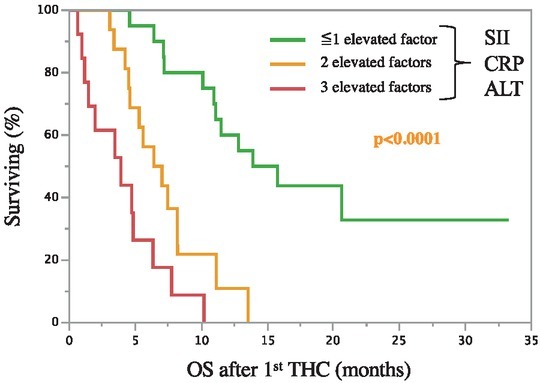
Scoring further improves overall survival estimation. Patients with zero to one elevated independent significant factors from multivariate analysis (C-reactive protein [CRP], systemic immune-inflammation index [SII], alanine aminotransferases [ALT]) had a significantly longer overall survival than patients with two or three elevated factors p < 0.0001.

**Table 3:** Scoring with significant independent factors from multivariate analysis. The number of elevated CRP, SII, ALT in patients further helps to predict median overall survival

Group	Median OS in months (95% CI)	Hazard ratio (95% CI)	p-value
≦ 1 elevated factor	14.9 (10.1–0.0)	0.08 (0.3–0.2)	-
2 elevated factors	6.7 (4.5–8.2)	0.38 (0.17–0.86)	**0.0003**
3 elevated factors	3.9 (1.15–6.3)	1	< 0.0001*

* = The difference between the of 2 and 3 elevated factors groups was statistically significant in univariate analysis, *p* = 0.022

## Discussion

Uveal melanoma patients with liver metastases have a grim prognosis with a median overall survival of 4–6 months and a 1-year survival rate of 12–15% when left untreated.^12^ Nowadays, thanks to treatment advances, the median overall survival after diagnosis of liver metastases is around 13.4 months, with a 2-year survival rate of 8%, which is similar to our study cohort.^13^, ^14^, ^15^, ^16^ As no treatment appears to be clearly superior over the others, identification of patients potentially benefitting from the treatment approach is vital for therapy allocation to provide the best care possible for each patient.^17^

Inflammatory cells in cancers have been thought to represent an antitumor response for many years. Nevertheless, there is a growing body of evidence that inflammation also plays a vital role in the initiation, malignization, and metastasis process of tumors driven by different immune cell subtypes.^7^, ^8^,^18^ In cancer patients, increased blood neutrophils and platelet counts have been associated with tumor progression and worse clinical outcomes in several solid tumors and can, therefore, be considered pro-tumorigenic.^19^, ^20^ Neutrophils achieve this by activating the endothelium and parenchymal cells through the secretion of soluble factors promoting adhesion to tumor cells at remote sites and thus promoting tumor spread.^21^, ^22^, ^23^ Platelets, by gathering around and thus also shielding tumor cells, promote adhesion, metastatic spread, and prevent cancer cell death.^20^

In contrast, lymphocytes play a crucial role in immuno-monitoring cancer by hampering tumor cell proliferation and migration by causing cytotoxic cell death.^24^ Thus, a high SII, comprising of elevated neutrophils and platelets and low counts of lymphocytes, suggests greater pro- than antitumorigenic activity with more unsatisfactory outcomes for patients. Not only could SII be identified as an independent prognostic factor for overall survival in this study, but it was also found to be statistically relevant in a recent meta-analysis on solid tumors.^25^

Similar to SII, an increased NLR could also be identified as a prognostic factor for overall survival in metastatic uveal melanoma disease.^26^, ^27^ Although NLR significantly predicted overall survival in the univariate analysis, it could not be confirmed as an independent. Considering the significant overlap and high correlation between SII and NLR, it is not unsurprising that only one remained significant in multivariate analysis, potentially emphasizing the additive value of incorporating platelet counts on the overall survival. Additionally, neutrophils also seem to be a relevant factor, as PLR was not significant in this analysis.

CRP, an acute-phase reactant reflecting tissue injury and inflammation, has long been suggested as a prognostic marker in several cancer types.^28^, ^29^ In a systematic literature review, elevated CRP was associated with higher mortality in patients with solid tumors in 90% of studies underlining the general prognostic relevance.^30^ In patients with metastatic uveal melanoma treated with immune checkpoint inhibitors showed that patients with high CRP had a significantly shorter survival in multivariate analysis (HR: 12.12; p = 0.001).^28^ Similarly, patients in this study succumbed significantly earlier when an elevated CRP before therapy was recorded.

Aside from immune markers, elevated Liver enzymes (AST and ALT) >ULN were identified as prognostic factors in univariate analysis for overall survival with ALT proving as independent as also by others in metastatic liver disease.^31^ It may be speculated that ALT and/or AST rise due to “space-occupying effects” associated with higher tumor burden, which could be confirmed in this patient set.

When stratified according to the numbers of elevated independent factors (CRP, SII, ALT), an even more distinct risk-based survival estimation may be achieved than just considering single factors.

Several study limitations should be acknowledged as enrollment of patients was retrospectively and limited to one institution with a treatment protocol and patient cohort characteristics that may substantially differ from other institutions hampering comparability to different patient cohorts. Moreover, the sample size did not allow for a validation cohort to confirm the results.

## Conclusions

In Patients treated with uveal melanoma liver metastases treated with transarterial chemoperfusion, lower pretreatment values of CRP, SII and ALT were independent prognostic factors associated with prolonged overall survival suggesting a role of systemic inflammation in this setting. Moreover, as the benefit of low pretreatment CRP, SII & ALT act synergistically, an even more distinct outcome stratification can be achieved. As physicians aim to provide the best possible care, the results from this study may help to identify patients who may benefit most from treatment or how best to stratify patients based on clinical risk factors in future clinical trials. Nevertheless, future prospective studies are warranted to confirm the relevance of pretreatment inflammatory markers and ALT for patient selection.

## References

[1] Carvajal RD, Schwartz GK, Tezel T, Marr B, Francis JH, Nathan PD (2017). Metastatic disease from uveal melanoma: treatment options and future prospects. Br J Ophthalmol.

[2] Singh AD, Turell ME, Topham AK (2011). Uveal melanoma: trends in incidence, treatment, and survival. Ophthalmology.

[3] Rietschel P, Panageas KS, Hanlon C, Patel A, Abramson DH, Chapman PB (2005). Variates of survival in metastatic uveal melanoma. J Clin Oncol.

[4] Leyvraz S, Piperno-Neumann S, Suciu S, Baurain JF, Zdzienicki M, Testori A (2014). Hepatic intra-arterial versus intravenous fotemustine in patients with liver metastases from uveal melanoma (EORTC 18021): a multicentric randomized trial. Ann Oncol.

[5] Mariani P, Dureau S, Savignoni A, Rouic LL, Levy-Gabriel C, Piperno-Neumann S (2019). Development of a prognostic nomogram for liver metastasis of uveal melanoma patients selected by liver MRI. Cancers.

[6] Lorenzo D, Piulats JM, Ochoa M, Arias L, Gutierrez C, Catala J (2019). Clinical predictors of survival in metastatic uveal melanoma. Jpn J Ophthalmol.

[7] Grivennikov SI, Greten FR, Karin M (2010). Immunity, inflammation, and cancer. Cell.

[8] Hanahan D, Weinberg RA (2011). Hallmarks of cancer: the next generation. Cell.

[9] Proctor MJ, Morrison DS, Talwar D, Balmer SM, O’Reilly DS, Foulis AK (2011). An inflammation-based prognostic score (mGPS) predicts cancer survival independent of tumour site: a Glasgow inflammation outcome study. Br J Cancer.

[10] Jomrich G, Hollenstein M, John M, Baierl A, Paireder M, Kristo I (2018). The modified glasgow prognostic score is an independent prognostic indicator in neoadjuvantly treated adenocarcinoma of the esophagogastric junction. Oncotarget.

[11] Ohno Y (2019). Role of systemic inflammatory response markers in urological malignancy. Int J Urol.

[12] Gragoudas ES, Egan KM, Seddon JM, Glynn RJ, Walsh SM, Finn SM (1991). Survival of patients with metastases from uveal melanoma. Ophthalmology.

[13] Krantz BA, Dave N, Komatsubara KM, Marr BP, Carvajal RD (2017). Uveal melanoma: epidemiology, etiology, and treatment of primary disease. Clin Ophthalmol.

[14] Diener-West M, Reynolds SM, Agugliaro DJ, Caldwell R, Cumming K, Earle JD (2005). Development of metastatic disease after enrollment in the COMS trials for treatment of choroidal melanoma: Collaborative Ocular Melanoma Study Group report No. 26. Arch Ophthalmol.

[15] Kujala E, Makitie T, Kivela T (2003). Very long-term prognosis of patients with malignant uveal melanoma. Invest Ophthalmol Vis Sci.

[16] Kuk D, Shoushtari AN, Barker CA, Panageas KS, Munhoz RR, Momtaz P (2016). Prognosis of mucosal, uveal, acral, nonacral cutaneous, and unknown primary melanoma from the time of first metastasis. Oncologist.

[17] Yang J, Manson DK, Marr BP, Carvajal RD (2018). Treatment of uveal melanoma: where are we now?. Ther Adv Med Oncol.

[18] Bronkhorst IH, Jager MJ (2013). Inflammation in uveal melanoma. Eye.

[19] Teramukai S, Kitano T, Kishida Y, Kawahara M, Kubota K, Komuta K (2009). Pretreatment neutrophil count as an independent prognostic factor in advanced non-small-cell lung cancer: an analysis of Japan Multinational Trial Organisation LC00-03. Eur J Cancer.

[20] Labelle M, Begum S, Hynes RO (2011). Direct signaling between platelets and cancer cells induces an epithelial-mesenchymal-like transition and promotes metastasis. Cancer Cell.

[21] Houghton AM, Rzymkiewicz DM, Ji H, Gregory AD, Egea EE, Metz HE (2010). Neutrophil elastase-mediated degradation of IRS-1 accelerates lung tumor growth. Nat Med.

[22] De Larco JE, Wuertz BR, Furcht LT (2004). The potential role of neutrophils in promoting the metastatic phenotype of tumors releasing interleukin-8. Clin Cancer Res.

[23] Chen HC, Lin HC, Liu CY, Wang CH, Hwang T, Huang TT (2004). Neutrophil elastase induces IL-8 synthesis by lung epithelial cells via the mitogen-activated protein kinase pathway. J Biomed Sci.

[24] Mantovani A, Allavena P, Sica A, Balkwill F (2008). Cancer-related inflammation. Nature.

[25] Zhong JH, Huang DH, Chen ZY (2017). Prognostic role of systemic immuneinflammation index in solid tumors: a systematic review and meta-analysis. Oncotarget.

[26] Nicholas MN, Khoja L, Atenafu EG, Hogg D, Quirt I, Butler M (2018). Prognostic factors for first-line therapy and overall survival of metastatic uveal melanoma: The Princess Margaret Cancer Centre experience. Melanoma Res.

[27] Zhan H, Ma JY, Jian QC (2018). Prognostic significance of pretreatment neutrophilto-lymphocyte ratio in melanoma patients: A meta-analysis. Clin Chim Acta.

[28] Heppt MV, Heinzerling L, Kahler KC, Forschner A, Kirchberger MC, Loquai C (2017). Prognostic factors and outcomes in metastatic uveal melanoma treated with programmed cell death-1 or combined PD-1/cytotoxic T-lymphocyte antigen-4 inhibition. Eur J Cancer.

[29] Mahmoud FA, Rivera NI (2002). The role of C-reactive protein as a prognostic indicator in advanced cancer. Curr Oncol Rep.

[30] Shrotriya S, Walsh D, Bennani-Baiti N, Thomas S, Lorton C (2015). C-Reactive Protein is an important biomarker for prognosis tumor recurrence and treatment response in adult solid tumors: A systematic review. PLoS One.

[31] Jiang Z, Li C, Zhao Z, Liu Z, Guan X, Yang M (2018). Abnormal liver function induced by space-occupying lesions is associated with unfavorable oncologic outcome in patients with colorectal cancer liver metastases. Biomed Res Int.

